# Study on the Preparation and Lipophilic Properties of Polyvinyl Alcohol (PVA) Nanofiber Membranes via Green Electrospinning

**DOI:** 10.3390/nano11102514

**Published:** 2021-09-27

**Authors:** Jun Cong Ge, Guirong Wu, Sam Ki Yoon, Min Soo Kim, Nag Jung Choi

**Affiliations:** Division of Mechanical Design Engineering, Jeonbuk National University, Jeonju-si 54896, Korea; jcge@jbnu.ac.kr (J.C.G.); wgr@jbnu.ac.kr (G.W.)

**Keywords:** polyvinyl alcohol, green electrospinning, engine oil, lipophilic properties, oil contact angle

## Abstract

As an environmentally friendly water-soluble polymer, polyvinyl alcohol (PVA) has attracted extensive attention because of its non-toxic, degradable, low cost, and good biocompatibility. Electrospinning is a kind of nanotechnology, and the nanofiber membrane prepared by it has the advantages of large surface area-to-volume ratios, nano- to micron-sized fibers, etc. Herein, a simple and facile one-step green electrospinning method was developed to fabricate various environmentally friendly PVA nanofiber membranes. The lipophilic properties of PVA membranes were investigated and optimized according different PVA concentrations. The PVA electrospun fiber prepared from the solution at a concentration of 10 wt% had the highest adsorption capacity for the adsorption of new and waste engine oils, and the waste engine oil adsorption capacity (12.70 g/g) was higher than that of new engine oil (11.67 g/g). It also has a relatively large BET surface area (12.05 m^2^/g), a pore volume (0.04 cm^3^/g), and an appropriate pore diameter (13.69 nm) and fiber diameter (174.21 nm). All electrospun PVA membranes showed excellent lipophilic properties due to their oil contact angles of much less than 30°. Therefore, PVA electrospun fibrous membranes have great application potential in the field of purifying engine oil due to the excellent lipophilic properties and oil absorption capacity.

## 1. Introduction

Electrospinning, as one of the simplest technologies to prepare nanofibers, has attracted wide attention for many years. Its working principle is that the spinnable polymer is subjected to an electric field force opposite to the direction of surface tension under the action of a high-voltage electrostatic field. When the electric field is strong enough, the spinning precursor is elongated from a spherical shape to a cone shape, that is, Taylor cone, so as to form continuous fibers with diameters ranging from 2 nm to several micrometers [1,2]. 

Compared with the traditional filtration membrane, electrospun nanofibrous membranes have large surface area-to-volume ratios, porous structure, and nano- to micron-sized fibers, which guarantee the excellent filtering performance [3,4]. The resulting as-spun nanofibers can also be modified by physicochemical methods (e.g., Ball milling and functional group modification) to obtain the desired porosity, pore sizes, and related functional groups [5,6,7]. 

Therefore, the electrospun fibers are widely used in filtration [8], ultrafiltration [9], affinity membranes [10], protective clothing [11], scaffolds in tissue engineering [12], enzyme immobilization [13], drug delivery [14], battery materials [15], sensor [16], and other fields [17]. The spinnable materials that are commonly used for electrospinning are polyurethane (PU) [18], polyacrylonitrile (PAN) [19], polyvinyl acetate (PVAc) [20], and polyvinylidene fluoride (PVDF) [21]. However, most of these commonly used polymers need to be dissolved in organic solvents, such as N, N-dimethylformamide (DMF), to prepare spinning precursors. DMF is a harmful solvent and raises severe concerns in production with respect to environmental and safety issues.

Therefore, it is necessary to find a new preparation method of nanofiber membranes. Recently, green electrospinning using an aqueous solution as the solvent has attracted great attention [22]. Polyvinyl alcohol (PVA) is a water-soluble polyhydroxy polymer, which is selected as the fiber precursor for filtering oil and has great application potential [23]. In recent years, using PVA as a hot spinning material has attracted substantial attention due to its cleanliness, safety, excellent chemical resistance, thermal stability, biodegradability, and the capability to be easily modified through its hydroxylic groups [24,25,26]. 

Pisuchpen et al. modified PVA fiber with multiple cycles of SiCl_4_/H2O treatment and silanization by electrospinning technology, which provides excellent water adhesion (the petal effect) [27]. Zhu et al. [28] mixed chitosan, SiO_2_ NPs, AgNO_3_, and PVA polymer, in turn, and finally prepared an environment-friendly multifunctional electrospun air filtration membrane with antibacterial properties and the ability to filter aerosol particles. 

Cui et al. [22] fabricated PVA/sodium lignosulfonate (LS) composite nanofiber membranes via green electrospinning. They reported that the addition of LS helped increase the PM2.5 removal efficiency compared to that of a pure PVA nanofiber membrane. Pervez et al. prepared the water soluble chitosan/polyvinyl alcohol (WSCHT/PVA) nanofiber at different mass ratios by using green electrospinning, and water was used as an ecofriendly solvent, and genipin was used as a nontoxic cross-linker. The composite membrane with 20/80 blend ratio had the most optimum uniform fiber morphology [29]. 

Other researchers have also studied the modifiable properties of PVA nanofiber membranes and optimized their ability to filter air and aqueous solution [30,31]. However, most studies focus on improving the hydrophilicity of the PVA electrospun membranes rather than enhancing the lipophilicity. The optimization of the lipophilic properties of PVA membranes is helpful to remove harmful substances in oil or realize oil–water separation [32].

To date, a pure PVA nanofibrous membrane has yet not been reported to have lipophilic properties according to the concentration of polymer. Therefore, this study mainly investigated the fabrication, characterization, and lipophilic properties of PVA membranes and optimized the PVA concentration on fiber morphology and lipophilic properties. An interesting result is that the PVA membrane exhibited excellent lipophilicity, which may enlarge the potential applications in the field of waste oil treatment.

## 2. Materials and Methods

### 2.1. Materials

Polyvinyl alcohol (PVA) with 80,000 g/mol of weight molecular weight (Mw) was used as the main material for fabricating the environmentally-friendly fiber membrane and was obtained from Hefei Sipin Technology Co., Ltd., Hefei, China. Pure water (conductivity: Max. 2.0 μs/cm; resistivity: Min. 0.5 MΩ) was used as the solvent and was procured from Samchun Pure Chemical Co., Ltd., Pyeongtaek-si, Korea. The oil samples included new and waste diesel engine oil, which were 5W30 synthetic oil, and their viscosities were 247.96 cP and 281.10 cP, respectively. The new and waste 5W30 synthetic engine oils were offered by Hyundai Oilbank Co., Ltd., Seoul, Korea.

### 2.2. Fabrication of the PVA Nanofiber Membrane

To observe the membrane-forming characteristics of the PVA fiber and obtain the best mixing ratio, a variety of PVA/water electrospinning solutions (8 wt%, 9 wt%, 10 wt%, 11 wt%, 12 wt%, 13wt%, 14 wt%, and 15 wt%) were prepared. The spinning process took about 8 h. The details are as follows: First, in order to obtain the initial spinning precursor of PVA, eight PVA powders with different mass fractions were respectively dissolved in the prepared pure water using a magnetic stirrer and dissolved for 24 h at room temperature. 

**Figure 1:**
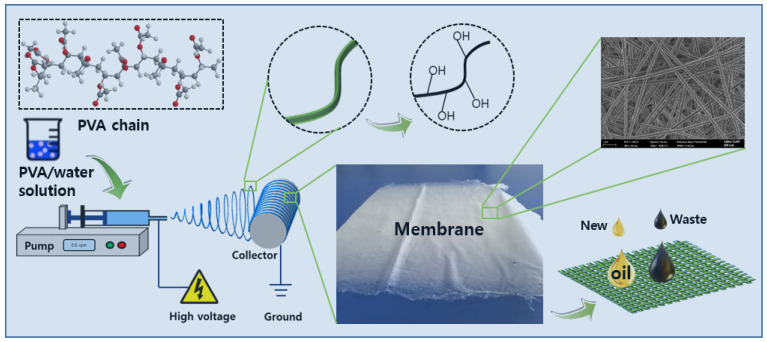
Schematic diagram of the electrospinning system.

Then, to obtain a homogeneous PVA spinning precursor, these initial spinning precursors were treated with ultrasonication for 2 h. Finally, the prepared PVA electrospinning solutions were injected into a 12 mL syringe with a 0.5 mm inner diameter needle (21G) to prepare for spinning. All as-prepared spinning solutions were electrospun under 20 kV of high-voltage electricity with a 100 mm tip-to-collector distance with a solution feed rate of 0.5 mL/h. The schematic diagram of electrospinning system is shown in Figure 1. 

### 2.3. Viscosity and Conductivity Measurements

The viscosities of the electrospun PVA solutions and oils were measured at 20 °C by a rotational viscometer (Viscolead One, Fungilab SA, Barcelona, Spain) using an L3-rotor at different rotational speeds (10–100 rpm). The conductivities of the electrospun solutions were determined at 20 °C by a digital conductivity meter (Starter 300C, Ohuas, Parippany, NJ, USA). The conductivity meter was pre-calibrated using conductivity standard solutions of 84, 1413, and 12,880 µS/cm at 298 K before starting to record data. All experiments were performed five times, and the average values were taken for the final results.

### 2.4. Characterization of the PVA Nanofiber Membrane

The surface structural morphologies of eight as-prepared electrospun PVA nanofiber membranes were observed with field emission scanning electron microscopy (FE-SEM, Supra 40VP, Carl Zeiss, Jena, Germany) at 2.0 kV and biological transmission electron microscopy (Bio-TEM, Hitachi Tem H-7650, Hitachi, Ltd., Tokyo, Japan) at 100 kV. The diameter distribution of the PVA fibers was measured by ImageJ software by measuring 50 randomly selected fibers. The structure and crystal phase of the as-prepared PVA fibrous membranes were analyzed by a multi-purpose high-performance X-ray diffractometer (XRD, PANalytical, Eindhoven, The Netherlands) with Cu Kα (λ = 1.540 Å) radiation. 

The chemical compositions of the as-prepared PVA fibrous membranes were analyzed by Fourier-transform infrared spectroscopy (FT-IR, Spectrum GX, PerkinElmer, Inc., Billerica, MA, USA) in transmittance, with a range of 400–4000 cm^−1^ and a resolution of 1 cm^−1^. The surface area, pore size, pore size distribution, pore volume, and other pore properties of the PVA membranes were measured using a surface area and ore size analyzer (BELSORP-max, MicrotracBEL Corp., Osaka, Japan) with nitrogen (N_2_) adsorption–desorption isotherms at 77 K under high vacuum. 

Before measuring the adsorption isotherms of N_2_ on the samples, the PVA membranes were degassed at 373 K for 24 h under vacuum to remove impurities on their surfaces. The multiple-point Brunauer–Emmett–Teller (BET) method and Horvaih–Kawazoe (HK) theory were employed to calculate the specific surface area and pore size distribution, respectively. The total pore volume was calculated at a relative pressure of P/P0 = 0.990.

### 2.5. Oil Adsorption Behavior Experiment

To investigate the adsorption behavior of the PVA membrane with different PVA concentrations on oil and explore the lipophilic properties, new and waste diesel engine oils were selected as the test objects. Respective 0.01 g samples of PVA membranes were put into a 50 mL falcon tube containing 20 mL of oil. The oil adsorption behavior experiment was carried out at 25 °C and 200 rpm in a shaking incubator (C-SKI, Chang Shin Scientific Co., Seoul, Korea) for 24 h. After adsorption for 24 h, the PVA membrane was removed from the oil bath and drained for 1 min to remove the residual oil on the material surface. The oil adsorption capacity of each PVA membrane was determined by the following equation [33]:

(1) $$Q=\frac{m_{a}-m_{0}}{m_{0}}$$

where *Q* (g/g) represents the oil adsorption capacity, *m_a_* is the total mass of the wet sorbent after 24 h of oil absorption followed by 1 min of draining, and *m*_0_ is the mass of the sorbent before the adsorption experiment. In order to reduce the error caused by the operations, each sample was measured at least three times.

### 2.6. Oil Contact Angle Measurement

The contact angle (CA) formed between the as-prepared electrospun PVA nanofiber membrane and oil was measured by a contact angle analyzer (UNI-CAM/M, GIT software Tech., Ansan-si, South Korea). The CA was determined by the Young−Laplace model and its value was averaged at six different locations in the sample using ImageJ software. The oil sorption behavior of the PVA membranes for new diesel engine oil and waste diesel engine oil can be simply and quickly investigated by observing the change of the oil contact angle.

## 3. Results

### 3.1. Physicochemical Properties of the Electrospun Fibrous Mat

**Figure 2:**
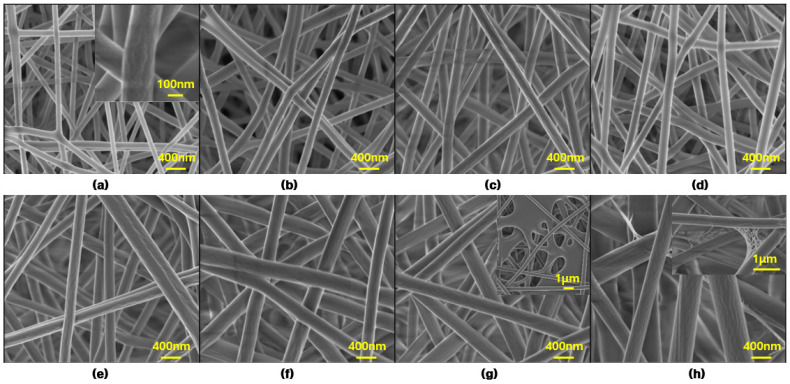
FE-SEM images of PVA fibers with various PVA solution concentrations: (**a**) 8 wt%, (**b**) 9 wt%, (**c**) 10 wt%, (**d**) 11 wt%, (**e**) 12 wt%, (**f**) 13 wt%, (**g**) 14 wt%, (**h**) 15 wt%.

To study the effect of PVA concentration on the fiber morphology and to optimize the mixing ratio, 8 wt%, 9 wt%, 10 wt%, 11 wt%, 12 wt%, 13 wt%, 14 wt%, and 15% PVA solutions were first electrospun. Figure 2 shows the field emission scanning electron microscopy (FE-SEM) images of the electrospun PVA fibers according to different PVA concentrations. As shown in Figure 2, the PVA fibers are arranged crosswise to each other, randomly distributed on the collected final mat and forming a non-woven network. Further, the PVA fiber surface does not seem to be very smooth due to the presence of some linear grooves (see Figure 2a, inset). 

These random crossover permutations form a large number of interconnected voids/porous structures between the fibers. The interconnected voids/porous structures are helpful for the adsorption effect because they provide more adsorption sites. In addition, it can be clearly seen that different PVA concentrations have a significant effect on the mean fiber diameters and other morphological characteristics. However, when the concentration of PVA exceeds 14 wt%, the nanofibrous structures of the PVA membranes are difficult to maintain due to their contraction, agglomeration, and adhesion between the fibers during the electrospinning process. 

A few spider-web-like structures (see Figure 2h, inset) are found in the 15 wt% PVA fibrous membranes. A high magnification shows that this spider-web-like fiber is grown from the main PVA nanofibers. The reason for the formation of this PVA spider-web-like structure may be related to the presence of PVA particles, because some PVA particles are not completely dissolved when excess PVA particles are added to water. This is consistent with the research results of other researchers [34]. The higher the viscosity of PVA spinning solution, the easier it is to form a spider web, as high viscosity may delay the thin film area to be evaporated properly.

Table 1 lists several important morphological characteristics of the PVA membrane, including the viscosity and conductivity of the PVA solution, fiber diameter, pore diameter, pore volume, and BET surface area. As shown in Table 1, the viscosity of the PVA solution clearly increases with the increase of the concentration of PVA in water, while the conductivity of the PVA solution shows a broadly increasing trend with increasing concentration. The thickness of PVA membrane increased with the increase of PVA concentration. 

The average fiber diameters were measured as 95.28 ± 12.05 nm, 126.07 ± 26.65nm, 174.21 ± 23.57 nm, 147.91 ± 20.93 nm, 190.66 ± 22.84 nm, 266.70 ± 30.61 nm, 308.92 ± 33.74 nm, and 479.46 ± 40.60 nm from the 8 wt%, 9 wt%, 10 wt%, 11 wt%, 12 wt%, 13 wt%, 14 wt%, and 15 wt% PVA solutions, respectively. The average fiber diameters and average pore diameters of eight PVA fibers ranged from 95 to 480 nm and 9 to 20 nm, respectively. These nanoscale fibers diameters and pore sizes are beneficial to increase the specific surface area of the fibers, thus, increasing the adsorption and adhesion ability of oil on the fiber surface [35]. 

With the increase of PVA concentration in water, the mean diameter of the PVA fiber first increased when the PVA concentration was between 8 wt% and 10 wt%, then decreased at 11 wt%, and increased again between 12 wt% and 15 wt%. This is mainly related to the comprehensive effect of viscosity and the conductivity of the PVA solution. The solution parameters, including viscosity and conductivity, play an important role regarding the fiber morphology (i.e., fiber diameter and structure) in the electrospinning process. In general, a higher viscosity and lower conductivity of the spinning solution hinder further stretching of the fiber, resulting in large-diameter nanofibers [36]. 

On the whole, in this study, the viscosity of PVA spinning solution plays a greater role than the conductivity in determining whether the fiber diameter becomes larger or smaller. This is because the increase of viscosity of PVA spinning solution is much greater than that of conductivity. The higher viscosity hinders the flow and elongation of spinning precursor, resulting in less stretched jet and increasing fiber diameter [37]. Moreover, different concentrations of the PVA spinning solution also have significant effects on the BET surface area and total pore volume in Table 1. 

The membrane containing 8 wt% PVA shows the highest BET surface area of 19.58 m^2^/g and a total pore volume of 0.06 cm^3^/g, while the 15 wt% PVA shows the lowest BET surface area of 5.06 m^2^/g and a total pore volume of 0.02 cm^3^/g. The change trend of bulk density is the same as that of BET. Although the decrease in fiber diameter is a factor to increase the fiber surface area, it can be concluded from the current results that variation of the BET surface area does not completely depend on the fiber diameter when the fiber diameter changes in a small range (<50 nm).

**Figure 3:**
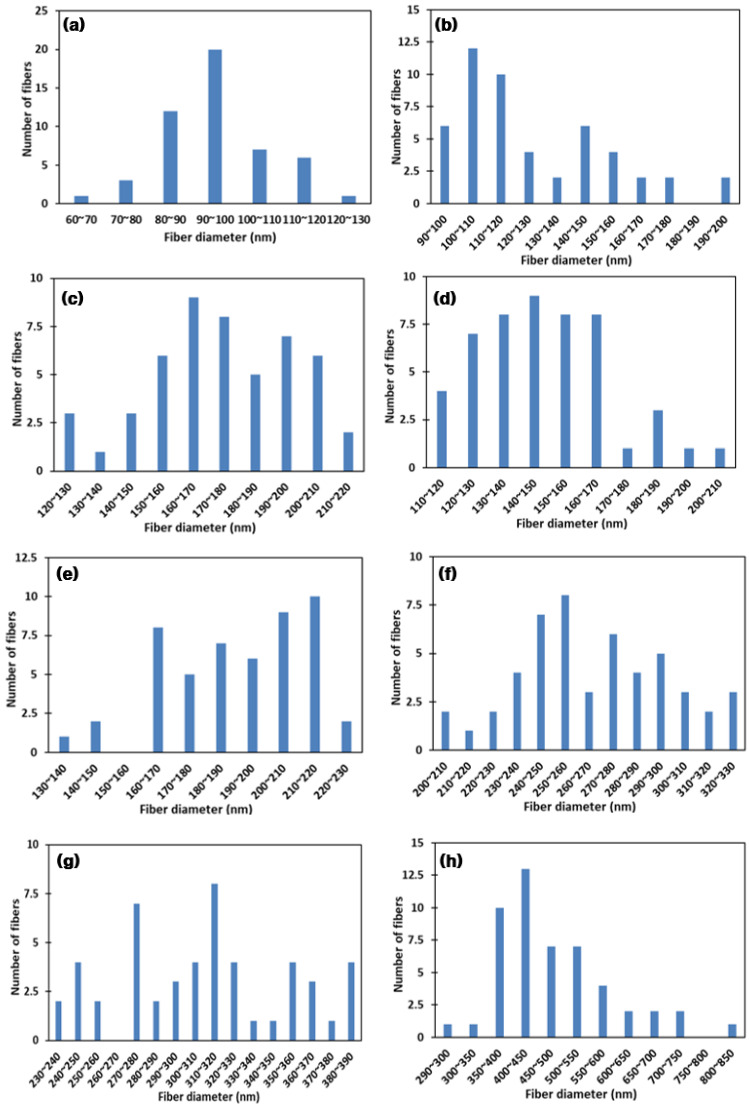
The fiber diameter distributions with various PVA solution concentrations: (**a**) 8 wt%, (**b**) 9 wt%, (**c**) 10 wt%, (**d**) 11 wt%, (**e**) 12 wt%, (**f**) 13 wt%, (**g**) 14 wt%, and (**h**) 15 wt%.

Figure 3 shows the PVA fiber diameter distribution according to different PVA concentrations. With an increase in PVA concentration, the average diameter of all fibers increased significantly compared with the 8 wt% sample. Most of the fiber diameters of the 8 wt% PVA are in the range of 90–100 nm, with a narrow diameter distribution. A somewhat broader distribution of other fiber diameters is observed when the PVA concentration exceeds 8 wt%. The 10 wt% and 11 wt% PVA fiber diameter distributions are more concentrated, in the range of about 120–180 nm. Overall, there is a nanometer-scale diameter distribution of all the PVA fibers, and the largest diameter of fibers does not exceed 850 nm. 

In this study, the concentration of PVA was used as the main variable, its influence on the viscosity and conductivity of the spinning precursor was investigated, and the fiber morphology was analyzed according to PVA concentration. In fact, in addition to the viscosity and conductivity of the spinning solution, some spinning parameters, such as applied voltage, tip-to-collector distance, flow rate, and needle diameter also affect the fiber morphology during the spinning process [38]. 

Appropriately increasing the voltage [39] and tip-to-collector distance [40], reducing the flow rate [41] are conducive to the stretching of spinning fiber and the evaporation of solvent to form small-diameter nanofibers. Most researchers have reported that the viscosity and conductivity of spinning precursors play the most important role in the influence of fiber morphology, compared with other spinning parameters [42]. Therefore, the operating parameters, such as applied voltage, tip-to-collector distance and flow rate, re optimized before the preparation of the PVA nanofiber membrane in this study.

**Figure 4:**
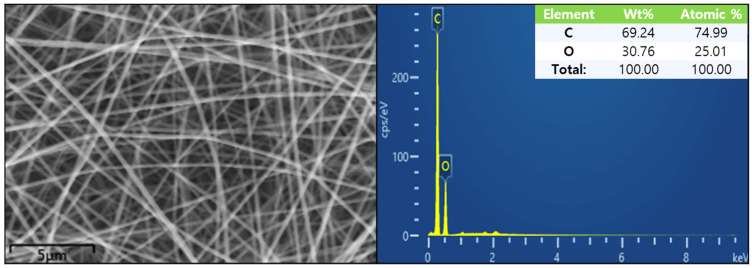
EDS spectra of PVA fibers prepared from the PVA solution at concentration of 10 wt%.

EDS spectra of a 10 wt% PVA membrane is shown in Figure 4. As shown in Figure 4, except for the signals of carbon and oxygen elements, no other signals of impurities were found, indicating that the PVA spinning solution was not polluted by the surrounding environment during the spinning process.

**Figure 5:**
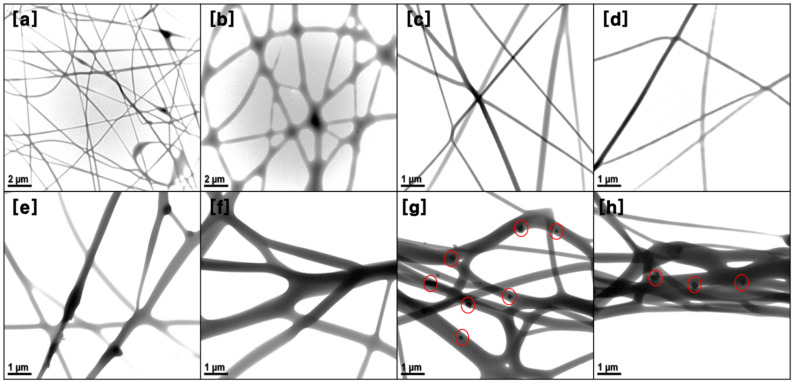
Bio-TEM images of PVA fibers with various PVA solution concentrations: (**a**) 8 wt%, (**b**) 9 wt%, (**c**) 10 wt%, (**d**) 11 wt%, (**e**) 12 wt%, (**f**) 13 wt%, (**g**) 14 wt%, and (**h**) 15 wt%.

Figure 5 shows the TEM images of a series of samples with different PVA concentrations. The PVA fibers are very homogenous in morphology and display a randomly distributed and interweaved to form a network. As shown in Figure 5a,b, some beaded fibers can be clearly observed when the concentration of the PVA spinning solution is low (i.e., 8 and 9 wt%). The fiber morphology is changed from a beaded fiber to a uniform fiber structure with increasing PVA concentration. 

This is mainly due to reduced chain entanglement in the spinning solution with a lower concentration, which leads to the contraction of the diameters of the jet and leads to jet instability of the polymer solution. Properly increasing the concentration of the spinning solution is beneficial to enhancing the stability of the polymer solution. However, the spinning effect is not very effective when the PVA concentration exceeds 14 wt%, as shown in Figure 5g,h. 

When the concentration of PVA exceeds 14 wt%, large diameter fibers are formed, fibers adhere together and many PVA particles appear in/on the surface of PVA fibers. This is because the inter- and intra-chain bonds between polar hydroxyl groups (-OH) of PVA molecules are not easily activated, which makes it difficult to form a liquid jet when the concentration of the spinning solution is too high [43]. Therefore, it is necessary to select the appropriate concentration of spinning solution for the formation of continuous and uniform fibers. Similar results were also reported by Zhang et al. [44]. They also indicated that, to obtain a large number of chain entanglements in the electrospinning process, the concentration of polymer solution must exceed a critical concentration.

**Figure 6:**
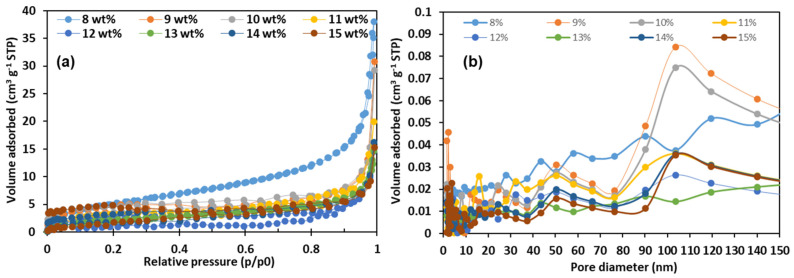
Nitrogen adsorption–desorption isotherms (**a**), and BJH adsorption pore size distribution (**b**) for all samples.

Figure 6 shows the nitrogen (N_2_) adsorption and desorption isotherm and Barret–Joyner–Halender (BJH) pore size distribution. The N_2_ adsorption-desorption method and BJH are common methods to investigate the pore structure and pore size distribution for fiber membrane materials. The pore structure characteristics of PVA nanofibrous membranes were obtained by the N_2_ adsorption-desorption measurements at 77 K. In Figure 6a, the adsorption isotherms of PVA membranes combine type I and type II characteristics, as there is a weak hystersis loop between the adsorption and desorption branches. 

In addition, it can be observed that these PVA membranes with low concentrations (<11 wt%) have large quantities adsorbed of N_2_, above 20 cm^3^/g, of which the highest value can reach up to 37.97 cm^3^/g at high relative pressure (P/P0) range (approaching 1.0), which indicates the existence of large mesopores and macropores [45]. In Figure 6b, the pore size distribution was calculated by the BJH method from the adsorption isotherm. As shown in Figure 6b, many peaks can be clearly observed, which indicates that the pore size distribution of PVA fiber membrane is not very uniform, including micropores (diameters < 2 nm), mesopores (diameters 2–50 nm), and macropores (diameters > 50 nm) [46].

### 3.2. XRD and FT-IR Analysis

**Figure 7:**
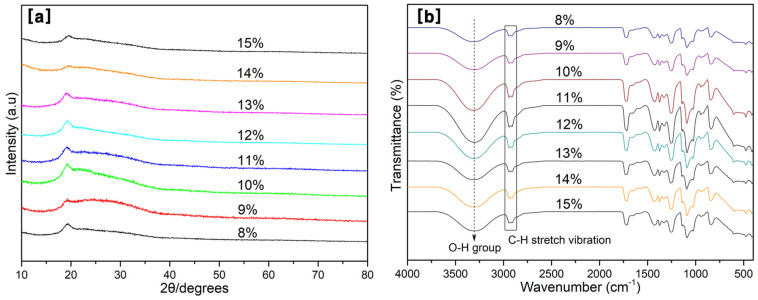
XRD (**a**) and FT-IR (**b**) analysis for all PVA membranes.

X-ray diffraction and FT-IR are often used to determine changes in microstructure and assess the chemical groups of polymers and their composites, respectively. Figure 7 depicts the XRD spectrum and FT-IR spectra for various fibers according to different PVA concentrations. As shown in Figure 7a, there is a strong diffraction peak appearing around 2θ = 19.2°, which is attributed to the (101) plane of PVA semicrystalline structures in the PVA fibers due to the occurrence of strong inter- and intra-molecular hydrogen bonding [47,48]. The intensity of this diffraction peak first increases and then decreases (from 13 wt% to 15 wt%) with the increase of PVA concentration. 

This may be due to the excessive addition of PVA, resulting in conditions under which the fiber cannot be well spun, forming more polymerization, thus, reducing the crystallinity of the PVA. Increasing the PVA content appropriately is beneficial to the crystallinity, which has been reported by other researchers [49]. The XRD results also support the fiber-forming properties of the PVA solution. Kurd et al. [48] reported that the crystal structure is related to the properties of the polymer (molecular weight), process parameters (applied voltage and flow rate), and solvent medium (evaporation rate and polymer-solvent interaction). 

As shown in Figure 7b, it can be clearly seen that the major characteristic peaks of the PVA membrane were observed at around 3305, 2943, 2910, 1716, 1420, 1371, 1146, 1092, and 838 cm^−1^. The peak at 3305 cm^−1^ is attributed to the O–H stretching vibration from the intermolecular and intramolecular hydrogen bonds, while the peaks at 2943 and 2910 cm^−1^ can be assigned to the –CH_2_– stretching vibration from methylene groups and the C–H stretching vibration from alkyl groups, respectively. 

The peak at 1716 cm^−1^ belongs to the C=O carbonyl stretch from acetate groups, while the peaks around 1420, 1371, 1146, 1092, and 838 cm^−1^ are assigned to the C–H bending vibration of CH_2_, C–H deformation vibration, C–O–C asymmetric stretching, C–O stretching of acetyl groups, and the C-C stretching vibration, respectively [50,51,52]. The specific wave number and vibration type are shown in Table 2. Similar to the results of XRD, the intensity of FTIR also shows a trend of first increasing and then decreasing with the increase of PVA concentration.

### 3.3. Oil Adsorption Capacity

**Figure 8:**
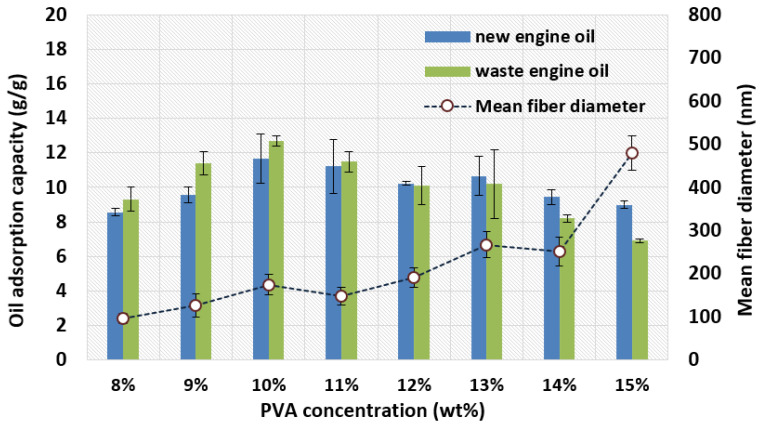
Oil adsorption capacities for various PVA membranes.

To investigate the oil adsorption capacity of all PVA membranes for the adsorption of new and waste diesel engine oils, the adsorption test was carried out at 25 °C and 200 rpm in a shaking incubator for 24 h. Figure 8 exhibits the oil adsorption capacity of all PVA membranes. The oil adsorption capacities of these PVA membranes were far less than those of an electrospun porous polystyrene (PS) membrane (about 900 g/g adsorption for oil) reported by [33]. Therefore, the optimized PVA membrane in this work has great potential to be applied to vehicle filters, such as fuel filters and oil filters. As a whole, the adsorption capacity of PVA membranes for the two oils increased first and then decreased with an increase of PVA concentration. 

The 10 wt% PVA membrane has the largest adsorption capacity for the two oils compared with other membranes. In addition, when the concentration of the PVA is between 8 wt% and 11 wt%, the adsorption capacity of the PVA membrane for waste engine oil is significantly greater than that for new engine oil. The adsorption capacities of the 12 wt% and 13 wt% PVA membranes for the two oils are almost the same. With the continued increase in the concentration of PVA to 14 wt% and 15 wt%, the adsorption capacity of the PVA membrane for two oils is reduced, and the adsorption capacity for new engine oil is higher than that for waste engine oil. 

Generally speaking, the thinner the fiber, the greater the porosity and BET surface area of the electrospun membrane, and the higher the adsorption capacity for the adsorption of the adsorbate. These advantages can provide more adsorption sites [53]. However, through the current research results, we found that, although the 8 wt% PVA membrane has the smallest fiber diameter (95.28 nm) and the largest pore volume (0.06 cm^3^/g) and BET surface area (19.58 m^2^/g) compared to other samples, the existence of a smaller pore diameter (11.84 nm) may be an important reason for preventing the penetration of larger oil molecules. 

A 9 wt% PVA membrane with a larger pore size of 19.87 nm can absorb oil droplets whose diameters are larger, but cannot retain oil droplets whose diameters are smaller than 19.87 nm, because small oil droplets pass through the membrane with large pore size [54]. Thus, a 10 wt% PVA membrane has a relatively large BET surface area (12.05 m^2^/g) and pore volume (0.04 cm^3^/g), and an appropriate pore size (13.69 nm) may be an important reason for its maximum oil adsorption capacity. 

Sarbatly et al. [55] also emphasized the importance of a proper pore size of polymer nanofibers for oil adsorption. In addition, the adsorption of these two oils by the PVA membranes is also related to the viscosity of the oil. After testing, the viscosities of the new and waste engine oils were 247.96 and 281.10 cP, respectively. When the PVA concentration is less than 11%, the PVA membranes have a smaller fiber diameter (<180 nm) and more easily promote the adhesion and penetration of high-viscosity oil due to the significant number of interconnected voids in the thinner fibers. By contrast, when the PVA concentration exceeds 11 wt%, the larger diameter fibers form larger gaps between fibers, resulting in lower oil sorption capacity. Similar results were reported by Wu et al. [35], Chen et al. [33], and Lin et al. [56].

Therefore, the adsorption mechanism of the PVA membrane on engine oil can be summarized as follows: (i) the existence of nanofibers makes the adsorption and capillary action dominant; (ii) the diameter and pore diameter of the fiber have a certain selectivity for oil adsorption, and the oils with higher viscosity more easily adhere to and penetrate into the surface of fibers with a porous structure and small diameter; (iii) the PVA membrane has excellent lipophilic properties, provides strong adhesion, and allows more oil droplets to approach the membrane surface; (iv) when the oil droplets contact with PVA membrane surface, the nanopores on each PVA fiber provide more space for oil adsorption, and the micropores between the fibers provide more channels for oil penetration; and (v) a PVA fibrous membrane has a porous structure and provides more volume for oil storage.

### 3.4. Oil Contact Angle

**Figure 9:**
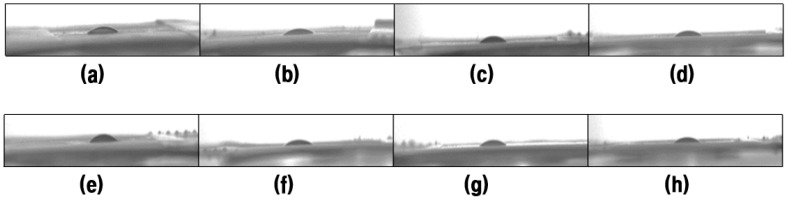
Optical micrographs of the new engine oil droplets; (**a**–**h**) represents the PVA solution concentration from 8% to 15%, respectively.

**Figure 10:**
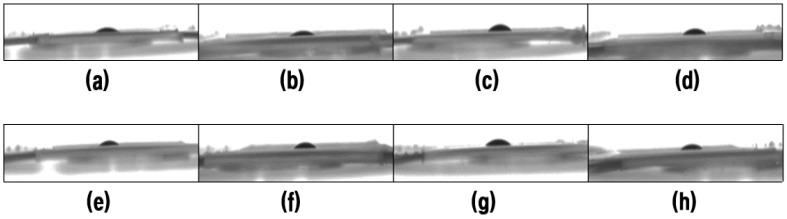
Optical micrographs of the waste engine oil droplets; (**a**–**h**) represents the PVA solution concentration from 8% to 15%, respectively.

**Figure 11:**
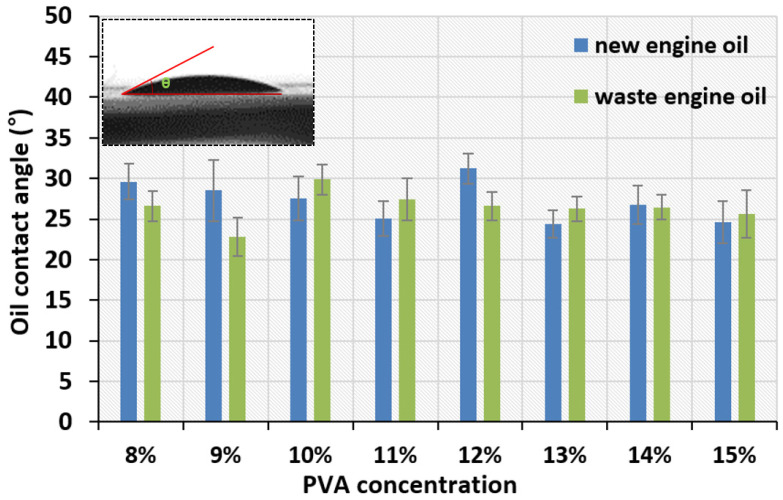
Oil contact angles for all tested PVA membranes.

To further study the lipophilic properties of the PVA membrane toward new and waste engine oils, at the 11th second after the oil droplet is dropped from the needle, captured by a high-speed camera, the contact angle between the oil droplet and the panel was measured with ImageJ software three times on each side. Selected optical micrographs of the oil droplets are shown in Figure 9, Figure 10 and Figure 11 for the variation of the oil contact angle with different PVA concentrations. 

As shown in Figure 11, all the oil contact angles are about 30° much less than 90°, which indicates that the electrospun PVA membrane has good lipophilicity. A superoleophilic surface is defined as a surface whose oil contact angle is close to 0° [23]. From Figure 11, the concentration of the PVA electrospinning solution has almost no influence on the contact angle value, because almost all the contact angles vary between 25° and 30°. A similar criterion was also proposed by [57]. However, when the PVA concentration exceeds 13 wt%, the oil contact angle of the PVA membrane seems to stabilize, showing a relatively low oil contact angle. 

The larger void spaces formed by large-sized fibers are beneficial to the initial penetration of oil. Moreover, the surface morphology and pore size of PVA nanofiber membrane also seems to play a crucial role in the influence of oil contact angle. 9 wt% and 15 wt% PVA nanofiber membrane have relatively large average fiber diameter, 19.87 nm and 17.30 nm, respectively. Therefore, most of oil droplets can easily pass through these large pores, resulting in relatively small oil contact angles. Other studies have also reported that the surface roughness of the nanofibers and air pockets is the main factor affecting the contact angle, and adding SiO_2_ nanoparticles into the spinning solution is beneficial to improve the fiber surface roughness [58,59].

## 4. Conclusions

In summary, eight different environmentally friendly polyvinyl alcohol (PVA) nanofibrous membranes were successfully fabricated via one-step electrospinning technology. To optimize the effect of the PVA concentration on the properties of the electrospun fibrous PVA membranes, the fiber morphology, the adsorption capacity of new and waste engine oils, and the oil contact angles were investigated and compared. Although the PVA membranes could be successfully prepared when the PVA concentrations were between 8 wt% and 15 wt%, undesirable beaded fiber and agglomerate structures were formed, respectively, when the PVA concentration was lower than 9 wt% or more than 14 wt%. 

The 10 wt% PVA membrane had the highest oil adsorption capacity for new and waste engine oils. The oil adsorption capacity of the PVA membrane mainly depended on physical adsorption, which had a direct relationship with the pore size and the BET specific surface area. The oil contact angle of almost all PVA membranes was lower than 30°, indicating that the PVA membranes had excellent lipophilic properties. Based on the fiber morphology, pore size, BET surface area, and oil adsorption capacity, the electrospun membrane containing 10% PVA performed the best.

## Figures and Tables

**Table 1:** Physical properties of the spinning solutions and electrospun fibrous membranes.

PVA Solutions (%)	Viscosity (cP)	Conductivity (μS/cm)	Mean Fiber Diameter (nm)	Mean Pore Diameter (nm)	Total Pore Volume (cm^3^/g)	BET Surface Area (m^2^/g)	Thickness (mm)	Bulk Density (cm^3^/g)
8%	241.34 ± 3.33	485.8 ± 1.92	95.28 ± 12.05	11.84	0.06	19.58	0.10	4.50
9%	457.24 ± 8.17	534.2 ± 1.10	126.07 ± 26.65	19.87	0.04	8.88	0.11	2.04
10%	655.4 ± 6.50	571.8 ± 5.17	174.21 ± 23.57	13.69	0.04	12.05	0.12	2.77
11%	1101.84 ± 16.82	608.2 ± 1.30	147.91 ± 20.93	13.08	0.03	9.31	0.18	2.14
12%	1602.94 ± 31.19	599 ± 4.18	190.66 ± 22.84	9.33	0.02	9.33	0.19	2.14
13%	2056.52 ± 33.73	674.6 ± 2.70	266.70 ± 30.61	11.01	0.02	7.93	0.20	1.82
14%	3615.84 ± 27.77	621.8 ± 1.30	308.92 ± 33.74	12.69	0.02	7.58	0.21	1.74
15%	5845.16 ± 69.42	663.8 ± 5.89	479.46 ± 40.60	17.30	0.02	5.06	0.22	1.16

**Table 2:** Main FTIR peaks for the PVA nanofiber membrane.

Wave Number (cm^−1^)	Vibration Type	Assignment
3305	stretching vibration	O–H
2943	stretching vibration	–CH_2_–
2910	stretching vibration	C–H
1716	carbonyl stretch	C=O
1420	bending vibration	C–H
1371	deformation vibration	C–H
1146	asymmetric stretching,	C–O–C
1092	stretching	C–O
838	stretching vibration	C-C

## Data Availability

Not applicable.
